# 10.Q. Workshop: Imbalance in use and impact of public psychotherapy – A Finnish working-age population register study

**DOI:** 10.1093/eurpub/ckac129.681

**Published:** 2022-10-25

**Authors:** Organised by: Finnish Institute of Occupational Health, Chair persons: Ari Väänänen (Finland), Sanna Selinheimo (Finland)

## Abstract

Mental disorders are globally among the leading causes of disability in the working-age population. Psychotherapies have proven to be effective in the improvement of mental health. The current evidence on the benefits of psychotherapies is mainly based on randomized control trials and patient-reported outcomes, whereas the potential impact of long-term psychological therapy on work disability and income is not so well known. This emphasizes the need for longitudinal studies on the effects of psychological therapies in a ‘real-world study setting’, and on the distribution of the availability and the use of psychological therapies in different populations. In Finland, rehabilitative psychotherapy is the major single form of publicly provided psychological therapy. It is targeted at those aged 16 to 67 who are at risk of disability or not being able to study because of mental health problems. It is granted an annual period (maximum three years), a maximum of 80 sessions per year and 200 sessions per 3 years. From 2011 on this psychotherapy has been statutorily granted to all at risk of work disability due mental health disorders. The number of annual users has increased from 15 757 (2010) to 56 682 (2020). This workshop presents the results from a research project investigating the use and real-world effects of long-term psychotherapy in the Finnish working-age population in the 2010s. It shows 1) whether the use of psychotherapy is associated with subsequent work disability and labour market outcomes (income, employment), 2) how the use of long-term psychotherapy differed between socio-demographic groups in the 2010s in Finland, and 3) the extent to which state-subsidized psychotherapy is linked to the distinctive profiles of mental health problems in the population. The five presentations offer new results from the Rise of Mental Vulnerability in Work Life Study drawn from the national registers. The presentations are mostly based on three randomly selected population cohorts which each included 33% of the 18-64-year-old permanent Finnish residents at the baseline. These data were sampled (sampling years: 2010, 2013, 2016) by and derived from the Population Register maintained by Statistics Finland and included information on various socio-demographic characteristics (e.g., gender, occupation class, region, income). These were linked to the national health registers. Information on reimbursed psychotherapy and mental health indicators (sickness absence, psychotropic drugs, disability benefits) were obtained from the Social Insurance Institution of Finland and complemented by the disability pension data from the Finnish Centre for Pensions. The data provide a unique opportunity to observe how the use of state-subsidized long-term psychotherapy was distributed across population groups in the 2010s, and to the extent to which disability trajectories and economic outcomes have developed in different groups during and after psychotherapeutic treatment.

**Key messages:**

• Psychotherapy is likely to decrease the risk of work disability and improve labour market outcomes.

• The provision of long-term psychotherapy is related to social inequality in the working population.

